# Participation of TFIIIB Subunit Brf1 in Transcription Regulation in the Human Pathogen *Leishmania major*

**DOI:** 10.3390/genes12020280

**Published:** 2021-02-16

**Authors:** Luis E. Florencio-Martínez, Andrés Cano-Santiago, Fabiola Mondragón-Rosas, Maricarmen Gómez-García, Carlos Flores-Pérez, Fiordaliso C. Román-Carraro, Luis A. Barocio-Rodríguez, Rebeca G. Manning-Cela, Tomás Nepomuceno-Mejía, Santiago Martínez-Calvillo

**Affiliations:** 1 Unidad de Biomedicina, Facultad de Estudios Superiores Iztacala, Universidad Nacional Autónoma de México. Av. de los Barrios 1, Col. Los Reyes Iztacala, Tlalnepantla, Edo. de Mexico, CP 54090, México; luisef@unam.mx (L.E.F.-M.); andres13cano@gmail.com (A.C.-S.); fabiiologa@gmail.com (F.M.-R.); mc.biologie@gmail.com (M.G.-G.); cflorespvip@gmail.com (C.F.-P.); fiordalisocrc@gmail.com (F.C.R.-C.); barocio.luis@gmail.com (L.A.B.-R.); tnepomuceno@unam.mx (T.N.-M.); 2 Departamento de Biomedicina Molecular, Centro de Investigación y de Estudios Avanzados del IPN, Av. IPN 2508, Ciudad de Mexico, CP 07360, México; rmanning@cinvestav.mx

**Keywords:** Pol III transcription, TFIIIB, *Leishmania*, tRNA, 5S rRNA, RNA polymerases

## Abstract

In yeast and higher eukaryotes, transcription factor TFIIIB is required for accurate initiation of transcription by RNA Polymerase III (Pol III), which synthesizes transfer RNAs (tRNAs), 5S ribosomal RNA (rRNA), and other essential RNA molecules. TFIIIB is composed of three subunits: B double prime 1 (Bdp1), TATA-binding protein (TBP), and TFIIB-related factor 1 (Brf1). Here, we report the molecular characterization of Brf1 in *Leishmania major* (LmBrf1), a parasitic protozoan that shows distinctive transcription characteristics, including the apparent absence of Pol III general transcription factors TFIIIA and TFIIIC. Although single-knockout parasites of LmBrf1 were obtained, attempts to generate LmBrf1-null mutants were unsuccessful, which suggests that LmBrf1 is essential in promastigotes of *L. major*. Notably, Northern blot analyses showed that the half-lives of the messenger RNAs (mRNAs) from LmBrf1 and other components of the Pol III transcription machinery (Bdp1 and Pol III subunit RPC1) are very similar (~40 min). Stabilization of these transcripts was observed in stationary-phase parasites. Chromatin immunoprecipitation (ChIP) experiments showed that LmBrf1 binds to tRNA, small nuclear RNA (snRNA), and 5S rRNA genes. Unexpectedly, the results also indicated that LmBrf1 associates to the promoter region of the 18S rRNA genes and to three Pol II-dependent regions here analyzed. Tandem affinity purification and mass spectrometry analyses allowed the identification of a putative TFIIIC subunit. Moreover, several proteins involved in transcription by all three RNA polymerases co-purified with the tagged version of LmBrf1.

## 1. Introduction

More than 20 different species of *Leishmania* produce the three main forms of leishmaniases (cutaneous, mucocutaneous, and visceral) in tropical and subtropical regions of the world. These protozoan parasites are transmitted to humans through the bite of infected female sandflies of the genera *Phlebotomus* and *Lutzomyia. Leishmania* and other trypanosomatids, such as *Trypanosoma cruzi* and *Trypanosoma brucei,* are ancient organisms that diverged early from the main eukaryotic lineage [1]. Consequently, their genes are expressed by atypical mechanisms, which include polycistronic transcription and trans-splicing [2,3,4].

Three distinctive classes of RNA polymerase (Pol) participate in transcription of nuclear DNA in eukaryotic cells: Pol I, II, and III [5]. Pol I is responsible for the synthesis of 18S, 5.8S, and 25–28S ribosomal RNAs (rRNAs) [6], whereas Pol II catalyzes the production of messenger RNAs (mRNAs), and most small nuclear RNAs (snRNAs) and microRNAs [7]. Pol III generates small essential noncoding RNA species, including transfer RNAs (tRNAs), 5S rRNA, U6 snRNA, and 7SL RNA [8,9]. Each Pol binds to specific promoter regions to initiate transcription. Promoters for Pol III are classified into three main types. The 5S rRNA genes possess type 1 promoters, which contain three gene-internal domains: box A, intermediate element, and box C. The tRNA genes contain internal type 2 promoters that consist of boxes A and B. Lastly, U6 snRNA genes have type 3 promoters composed of three small domains positioned upstream of the transcription start site: TATA box, proximal sequence element, and distal sequence element [10]. 

In yeast and higher eukaryotes, precise initiation of transcription by Pol III requires general transcription factors TFIIIA, TFIIIB, and TFIIIC [11]. TFIIIB interacts with all three types of promoter regions, where it is needed for the assembly of the transcriptionally active preinitiation complex (PIC), given that it recruits and positions Pol III to the start site and is involved in promoter opening [12]. TFIIIB is composed of three subunits: the TATA box-binding protein (TBP), B double prime 1 (Bdp1), and the TFIIB-related factor 1 (Brf1) [13]. In vertebrates, Brf2 replaces Brf1 at type 3 promoters [14]. Notably, the Brf gene family has three members in *Arabidopsis* (Brf1, Brf2, and Brf3) [15]. 

Brf1 (and all the Brf family members) is structurally and functionally related to Pol II transcription factor TFIIB and to subunit TAF1B (known as Rrn7 in yeast) of transcription factor SL1 (called Core Factor in yeast) of Pol I [16]. They all possess a zinc ribbon motif and two cyclin repeats [17]. Brf1 has a C-terminal extension, not present in TFIIB, which contains three sequence domains, known as Brf1 homology blocks I–III, which are conserved in several yeast species and human [18]. The N-terminal region of Brf1 interacts directly with subunits RPC1 (C160), RPC2 (C128), RPC3 (C82), RPC6 (C34), and RPC9 (C17) from Pol III, as well as with subunits Tau131, Tau95 and Tau60 from TFIIIC [18,19,20,21,22,23,24,25]. On the other hand, the Brf1 C-terminal region is involved in interactions with TBP and Bdp1 [18,23,24]. 

The knowledge about Pol III transcription in *Leishmania* spp. is scarce. Notably, Pol III synthesizes all snRNAs (not only U6) in these organisms, in addition to tRNAs and 5S rRNA [26,27]. While tRNAs and 5S rRNA genes seem to contain archetypal internal promoter sequences (boxes A and B; boxes A and C and intermediate element, respectively) [28,29], expression of snRNA genes is directed by atypical external and internal promoter elements [30]. For instance, transcription of the U2 snRNA gene in *L. major* is dependent on four different regions: boxes A and B from a neighbor tRNA-like, box B from an upstream tRNA-Ala gene, and an intragenic promoter element located close to the 5′ end of the U2 snRNA gene [27]. 

Interestingly, out of the three general transcription factors required by Pol III to initiate transcription, only TFIIIB has been identified in *Leishmania*. Although subunit TBP (which is shared by all Pol) has been mainly studied in the context of Pol II transcription, it has been demonstrated that it binds to tRNA and snRNA genes in *L. tarentolae* and *L. major* [31,32]. The SNAP50 subunit of the SNAP complex was also found to associate with tRNA and snRNA genes [32]. A recent report indicates that TFIIIB subunit Bdp1 is an essential protein required for Pol III transcription of tRNAs, snRNAs, and 5S rRNA in *L. major* [33]. In *T. brucei*, tandem affinity purification experiments with a Prot C-TEV-Prot A (PTP)-tagged version of TBP demonstrated that Brf1 co-purified with TBP [34]. By generating Brf1 conditional knockdown cell lines, it was shown that Brf1 is needed for cell viability and participates in Pol III transcription in *T. brucei* procyclic forms [35]. 

Here, we characterize the Brf1 ortholog in *L. major* (LmBrf1). Bioinformatic analyses showed that LmBrf1 contains semiconserved homology blocks I–III, characteristic of Brf1 orthologs in yeast species and human. Unsuccessful attempts to generate LmBrf1 double-knockout parasites suggest that LmBrf1 is essential in promastigotes of *L. major*. Our results also indicate that the mRNAs of LmBrf1 and two other proteins involved in Pol III transcription show similar decay rates. Chromatin immunoprecipitation (ChIP) assays demonstrated the in vivo association of LmBrf1 with tRNA, snRNA, and 5S rRNA genes. Unexpectedly, our data suggest the association of LmBrf1 with the Pol I promoter of the rRNA transcription unit and with three genomic regions transcribed by Pol II that were here examined. Moreover, tandem affinity purification and mass spectrometry analyses identified proteins involved in transcription by all three RNA polymerases as putative interacting partners of LmBrf1.

## 2. Materials and Methods

### 2.1. Bioinformatic Analyses

Protein sequences were obtained from the TriTrypDB database (release 47) (http://tritrypdb.org/tritrypdb/) or the National Center for Biotechnology Information (NCBI) database (http://www.ncbi.nlm.nih.gov). Sequence alignments were generated with the Clustal Omega program (http://www.ebi.ac.uk/Tools/msa/clustalo/) and shaded manually. Secondary structure predictions were obtained with the PSIPRED Protein Sequence Analysis Workbench (http://bioinf.cs.ucl.ac.uk/psipred/, accessed on 1 March 2020). Homology modeling was performed with the UCSF Chimera program (https://www.cgl.ucsf.edu/chimera/, accessed on 1 March 2020) [36], using the crystallographic structure of Brf1 from yeast (Protein Data Bank (PDB) identifier (ID): 6eu0) as a template. The quality of the models was evaluated with the Mod Eval program (https://modbase.compbio.ucsf.edu/evaluation/, accessed on 1 January 2021). Some of the proteins identified by mass spectrometry were analyzed with the HHpred server (https://toolkit.tuebingen.mpg.de/tools/hhpred, accessed on 1 December 2020) [37].

### 2.2. Cell Culture and Transfection

*L. major* promastigotes from strain MHOM/IL/81/Friedlin (LSB-132.1) were grown in BM medium (1× M199 medium pH 7.2 containing 10% heat-inactivated fetal bovine serum, 9.5 g/L brain heart infusion, 40 mM HEPES, 0.01 mg/mL hemin, 0.0002% biotin, 100 IU/mL penicillin, 100 μg/mL streptomycin, and 1× l-glutamine) at 26 °C and harvested in the mid-logarithmic or stationary phase. Transfection of promastigotes with the episomal LmBrf1-PTP vector was performed by electroporation as previously described [33]. Briefly, 4 × 10^7^ promastigotes in 0.4 mL of electroporation buffer (25 mM HEPES, 120 mM KCl, 0.15 mM CaCl_2_, 5 mM MgCl_2_, 10 mM KH_2_PO_4_, 10 mM K_2_HPO_4_, and 2 mM EDTA, pH 7.6) were transfected with 10 μg of circular plasmid DNA by electroporation at 1600 V, 50 μF, and 25 Ω (BTX Electro Square Porator ECM 830). Cells were grown for 24 h before spreading on plates containing 0.7% Seaplaque GTG agarose (FMC Bioproducts, Philadelphia, PA, USA) in BM medium with 50 μg/mL G418. Some of the isolated colonies were selected for further analysis. The same electroporation conditions were used for transfections with the *pac* and *hyg* targeting cassettes, for the generation of knockout parasites.

### 2.3. Generation of Plasmids 

To produce vector pΔBrf1-pac for knockout experiments, the *pac* gene (1.7 kb) was obtained by digesting the pΔ75-pac plasmid [38] with *Eco*RI and *Sac*I. The 5′-targeting region from LmBrf1 (*LmjF.25.0440*) (531 bp) was amplified by PCR with primers BRF1-5-for-*Xba*I (5′–ATCTAGAACGGCACACATCTACTCGCG) and BRF1-5-rev-*Eco*RI (5′–AGAATTCGGTGAAGCGTCAACTGCTGG). The 3′-targeting region (508 bp) was amplified with oligonucleotides BRF1-3-for-*Sac*I (5′–AGAGCTCCGAATACGCTGCCAGAGTAG) and BRF1-3-rev-*Xho*I (5′–ACTCGAGAGCTTTCGTCTCTTAGGCCC). To generate vector pΔBrf1-hyg, the *hyg* gene (1.8 kb) was obtained from the pΔ75-hyg plasmid [38] after *Spe*I and *Sac*I digestion. The 5′-targeting region was amplified with primers BRF1-5-for-*Xba*I and BRF1-5-rev-*Spe*I (5′–AACTAGTGGTGAAGCGTCAACTGCTGG), and the 3′-targeting region with oligonucleotides BRF1-3-for-*Sac*I and BRF1-3-rev-*Xho*I. The PCR products from 5′ and 3′ targeting regions were digested with the restriction enzymes indicated in the name of each oligonucleotide. For both knockout vectors, the selectable-marker gene and the two flanking regions were cloned into pBluescript II KS (digested with *Xba*I and *Xho*I), by a single four-fragment ligation step. The correct order of the fragments was verified by sequencing. The targeting cassettes were obtained from pΔBrf1-pac and pΔBrf1-hyg by digesting with *Xba*I and *Xho*I. To generate the LmBrf1-PTP vector, for immunofluorescence, tandem affinity purifications, and ChIP assays, the RPB6 gene from the pB6-PTP plasmid [29] was substituted with the LmBrf1 gene. For this purpose, pB6-PTP was digested with *Xma*I and *Xba*I, and the RBP6 gene was eliminated by agarose gel electrophoresis. The LmBrf1 gene (without the terminal codon) was amplified with primers BRF-*Xma*I-5′ (5′–ATCCCGGGATGTCCAGCTGCACCCATCCCA) and BRF-*Xba*I-3′ (5′–ATTCTAGAGTCCCAGTCCGCCTGTGGCTCA). The PCR product was first cloned into the pGEM-T Easy vector (Promega, Philadelphia, PA, USA), digested with *Xma*I and *Xba*I, and then ligated into the pB6-PTP backbone. The vector was sequenced to confirm that the LmBrf1 gene and the tag were in frame.

### 2.4. Indirect Immunofluorescence Assays 

The subcellular localization of LmBrf1 labeled with a PTP tag was determined by indirect immunofluorescence, as previously described [29,39]. Parasites fixed with paraformaldehyde were stained for double indirect immunofluorescence analysis using a mixture of primary antibodies against Prot C (Delta Biolabs, Gilroy, CA, USA) (to detect LmBrf1-PTP) and LmNop56, followed by anti-rabbit immunoglobulin G (IgG) conjugated with Alexa Fluor 568 (red) and anti-mouse IgG coupled with Alexa Fluor 488 (green) secondary antibodies. DNA was stained with 4’,6-diamidino-2-phenylindole (DAPI) (blue). Individual optical sections were obtained with a Nikon A1R+ STORM confocal microscope, analyzed, and prepared for presentation using the ZEN 2012 software (Blue edition).

### 2.5. Western Blot Analysis

Whole-cell protein extracts were obtained by standard protocols [40]. Briefly, 2 × 10^8^ parasites were lysed in RIPA buffer (150 mM NaCl, 5 mM EDTA, 0.5% sodium deoxycholate, 0.1% SDS, 50 mM Tris, pH 7.4, and 1% Nonidet P-40 (NP-40)) and 1× protease inhibitors (Sigma, Saint Louis, MI, USA). The extract was incubated for 30 min on ice and mixed every ten minutes, and then centrifuged at 15,000× *g* for 20 min. The supernatant was recovered and stored at −70 °C. For Western blot analysis, 50 µg of total protein was fractionated by 10% SDS-PAGE and blotted onto polyvinylidene difluoride (PVDF) membranes (Bio-Rad, Hercules, CA, USA). Membranes were incubated with rabbit primary antibodies against Prot C (1:13,000 dilution, Delta Biolabs, Gilroy, CA, USA) or polyclonal human α/β-tubulin antibody (1:1000, Cell Signaling Technology, Danvers, MA, USA). Proteins were detected with a horseradish peroxidase (HRP)-conjugated secondary antibody and developed using the Immobilon Western Chemiluminescent HRP substrate (Millipore, Burlington, VE, USA).

### 2.6. Chromatin Immunoprecipitation Assays

The ChIP procedures were performed as previously described [41]. Briefly, 1.2 × 10^8^ promastigotes were cross-linked with formaldehyde (final concentration of 1%) for 5 min at 37 °C. A Vibra-Cell VCX130 ultrasonic processor (Sonics, Oklahoma City, OK, USA) was employed to lyse the cells (15 s on/off, 40% amplitude, for 5 min). Nuclei were pelleted and resuspended in sonication buffer (1% SDS, 10 mM EDTA, and 50 mM Tris-HCl, pH 8.0; 1× protease inhibitors). Chromatin was sonicated to an average DNA size of about 200 to 500 bp with a BioRuptor UCD-200 (Diagenode, Denville, NJ, USA) (30 s on/30 s off, high intensity) for 30 cycles. The sonicated material was pre-cleared by adding protein A/G plus-agarose beads (Santa Cruz Biotechnology, Dallas, TX, USA) and mixing for 1 h at 4 °C. Chromatin samples were incubated overnight at 4 °C with rabbit anti-Prot A antibody (Sigma, Saint Louis, MI, USA) or nonspecific rabbit immune serum (negative control). Protein–DNA complexes were incubated for 2 h with protein A/G plus-agarose beads and 20 µg of sonicated salmon sperm DNA, and then washed as previously described [42]. Cross-links were reversed with 200 mM NaCl at 65 °C overnight. Samples were treated with RNase A and proteinase K. DNA was precipitated with sodium acetate and ethanol and quantified. Each ChIP experiment was performed at least three times.

### 2.7. Quantitative Real-Time PCR Experiments

Around 5 ng of immunoprecipitated DNA was employed to examine by quantitative real-time PCR with the Platinum SYBR Green qPCR Super Mix-UDG kit (Invitrogen, Carlsbad, CA, USA). The results were analyzed using the Ct method, as reported previously [35,42]. Reactions were carried out with optimized conditions that produce a single amplicon of the correct size, in duplicate. Results are presented as percentage of input, corrected by subtracting corresponding values from negative control precipitations performed with a nonspecific antiserum. The tRNA-Ala gene (*LmjF.31.TRNAALA.01*) was amplified with primers tRNA-Ala-5′ (5′–ATTGGGACGTTACCGCGTCG) and tRNA-Ala-3′ (5′–ATTGCGGCCCAGGCCTTTCA). The tRNA-like associated to the U2 snRNA gene was amplified with primers U2tRNA-like-5′ (5′–CCGAGAAGATATGTTAGTACCACC) and U2tRNA-like-3′ (5′–AGGAAAAGATGCTTTCGACGAG), and the U2 snRNA gene (*LmjF.31.snRNA.01*) with oligonucleotides U2-5′ (5′–AAACGTGGAACTCCAAGGAA) and U2-3′ (5′–TATCTTCTCGGCTATTTAGC). The U4 snRNA (*LmjF.36.snRNA.01*) was amplified with primers U4-5′ (5′–AAGCCTTGCGCAGGGAGATGT) and U4Lmjend-XbaI-R (5′–ATCTAGAGACAAAAAGTGTTCCCCACC), and the tRNA-like associated to the U4 snRNA with oligonucleotides U4tRNA-like 5′ (5′–GAAAAAAGGAGCGCCGCCCCA) and U4tRNA-like 3′ (5′–CGCAAGGCTTGCCTTGGGTGT). The 5S rRNA gene (*LmjF.11.5SRRNA.03*) was amplified with primers 5SrRNA-F1 (5′–GAGTACGACCACACTTGAGTG) and 5SrRNA-R1 (5′–GAGTACGGCACTCAGGGTT), and the tRNA-Met gene (*LmjF.11.TRNAMET.01*) with primers tRNAmet-F (5′–AAAGTTTGCGACCGGTGAG) and tRNAmet-R (5′–CACAACTTTCACTCGTAGCCG). The intergenic region fragment (Inter 11.0930) located between *LmjF11.0930* and the 5S rRNA gene was amplified with primers 11-Inter-5′ (5′–GAACTTGGGAATGCCTTCTG) and 11-Inter-3′ (5′–GCAAGAAGAATGTGGAACGG), and the *LmjF11.0930* gene with primers 11.0930-5′ (5′–AGCAGCAGTTCATTGAGGCT) and 11.0930-3′ (5′–GCCGATCATCATCCTCTAAG). The fragment from the strand switch region from chromosome 1 (SSR-Chr1) was amplified with oligonucleotides ssr4-F (5′–AATCACAGCACGCATACACG) and ssr4-R (5′–GCGTCATGGCTTCACTAACAG). The promoter (SL prom) of the spliced-leader RNA gene (*LmjF.02.SLRNA.0010*) was amplified with oligonucleotides LmjF-SL-promF (5′–GAGCGCGGTGGGCATGACA) and LmjF-SL-promR (5′–AAGCCATCACCACCGCAGC), and the intergenic region from the spliced-leader locus (Inter SL) with primers LmjF-SL-InterF (5′–TGTGCGTGCGTGTGGTGGT) and LmjF-SL-InterR (5′–CGGGCGCACCCTTGCAGT). The promoter region of the rRNA transcription unit (18S prom) was amplified with primers rRNA-A-5′ (5′–TTGTTTGGGTGGAGGTGAGA) and rRNA-A-3′ (5′–CAAAATCATCAAACCCGTTC), and the 18S rRNA gene (*LmjF.27.rRNA.01*) with primers rRNA-18S-5′ (5′–CATGCATGCCTCAGAATCAC) and rRNA-18S-3′ (5′–CGTTTCGCCAAGTTATCCAA).

### 2.8. Northern and Southern Blot Analyses

For determination of the half-life of the different mRNAs, Northern blot experiments were performed with RNA isolated at several time points from cells incubated in the presence of the transcription inhibitor actinomycin D (10 µg/mL). Total RNA was extracted with TRI reagent (Sigma, Saint Louis, MI, USA), separated in agarose–formaldehyde gels and transferred to Hybond-N filters (Amersham, v). Hybridization was performed with probes that were labeled with [α-^32^P]dCTP using the High Prime labeling system (Amersham, Little Chalfont, UK). The Brf1 probe was amplified with primers BRF-*Xma*I-5′ and BRF-*Xba*I-3′. The Bdp1 probe was amplified with oligonucleotides B’’-*Bam*HI-5′ (5′–ATGGATCCATGGACGACAACGAGTTCGAA) and B’’-*Xba*I-3′ (5′–ATTCTAGACTCAAACGAGAAGTCCGAGTC) and the RPC1 probe with primers C160-1-5′ (5′–GATCATCAACGCCAACAAGAGC) and C160-2-3′ (5′–TTGAGCAGGATGTTGAAGCTGC). The α-tubulin probe was amplified with primers Alfa-tub-5′ (5′–AGAAGTCCAAGCTCGGCTACAC) and Alfa-tub-3′ (5′–GTAGTTGATGCCGCACTTGAAG), and the 18S rRNA probe with oligonucleotides Lm-rRNA18S 5′ (5′–CGGCCTCTAGGAATGAAGG) and Lm-rRNA18S 3′ (5′–CCCCTGAGACTGTAACCTC). Filters were washed to a final stringency of 0.1× SSC and 0.1% SDS at 65 °C. RNA signals from three independent experiments were quantified by densitometry using the MultiGauge software and plotted. Half-lives were determined from the linear regression estimates derived from the linear segment of the mRNA decay curves. For Southern blot analysis, 5 µg of genomic DNA was digested, separated by electrophoresis, and transferred by capillary to Hybond-N membranes. The radioactive probe corresponded to the 5′-targeting region of Brf1. Filters were washed as described above.

### 2.9. Tandem Affinity Purifications and Mass Spectrometry Analysis

Tandem affinity purification experiments were performed, as previously described [43,44], using whole-cell extracts obtained from mid-log phase promastigotes (3 L at 3–4 × 10^7^ cells per mL). Cells were harvested, washed with phosphate-buffered saline (PBS), and resuspended in 14 mL of IPP-150 buffer (10 mM Tris–HCl, pH 8.0, 150 mM NaCl, and 0.1% NP-40) that contained two tablets of Complete Mini, EDTA-free protease inhibitor cocktail (Roche Molecular, Basel, Switzerland). Then, 2 mL of 10% Triton X-100 was added and the samples were maintained on ice for around 20 min, until cells were totally lysed. To obtain the cleared lysate, samples were centrifuged at 10,000× *g* for 15 min at 4 °C. Then, the extract was added to a 20 mL disposable column, in which 200 µL of IgG Sepharose 6 Fast Flow beads (GE Healthcare, Chicago, IL, USA) was equilibrated with IPP-150 buffer and incubated with rotation for 3 h at 4 °C. The column was drained by gravity flow and washed three times with 20 mL of cold IPP-150 buffer. PTP-tagged proteins were eluted by resuspending the beads in 2 mL of TEV cleavage buffer (20 mM Tris–HCl, pH 7.7, 150 mM KCl, 3 mM MgCl_2_, 0.5 mM dithiothreitol (DTT), 0.1% Tween-20, and 0.5 mM EDTA) with 200 U of tobacco etch virus (TEV) protease (Sigma), and incubating overnight at 4 °C. The IgG-Sepharose was drained and the eluted material was mixed with 4 mL of PC-150 buffer (20 mM Tris–HCl, pH 7.7, 150 mM KCl, 3 mM MgCl_2_, 0.5 mM DTT, 0.1% Tween-20, and 2× protease inhibitors). CaCl_2_ was added to the eluate to a final concentration of 3 mM before transfer to a 10 mL column containing 200 µL of anti-Prot C affinity matrix (Roche, Basel, Switzerland). Samples were incubated at 4 °C for 4 h with rotation, washed with 30 mL of PC-150, and Prot C-tagged proteins were eluted with EGTA elution buffer (5 mM Tris-HCl pH 7.7, 10 mM EGTA, 5 mM EDTA, and 2× protease inhibitors). Eluted proteins were concentrated with Amicon Ultracel 3K columns (Millipore) or by evaporation in a vacuum concentrator and analyzed by SDS–PAGE and SYPRO Ruby (Molecular Probes, Eugene, OR, USA) staining. Individual lanes from the gels were sliced into two pieces and proteins subjected to in-gel tryptic digestion prior to liquid chromatography–mass spectrometry/mass spectrometry (LC–MS/MS) at the Core Facility for Proteomics and Mass Spectrometry from Upstate Medical University. The collision-induced dissociation (CID) spectra were compared with the *L. major* protein database from TriTrypDB page.

## 3. Results

### 3.1. The Sequence and Predicted Structure of LmBrf1 Are Conserved

**Figure 1:**
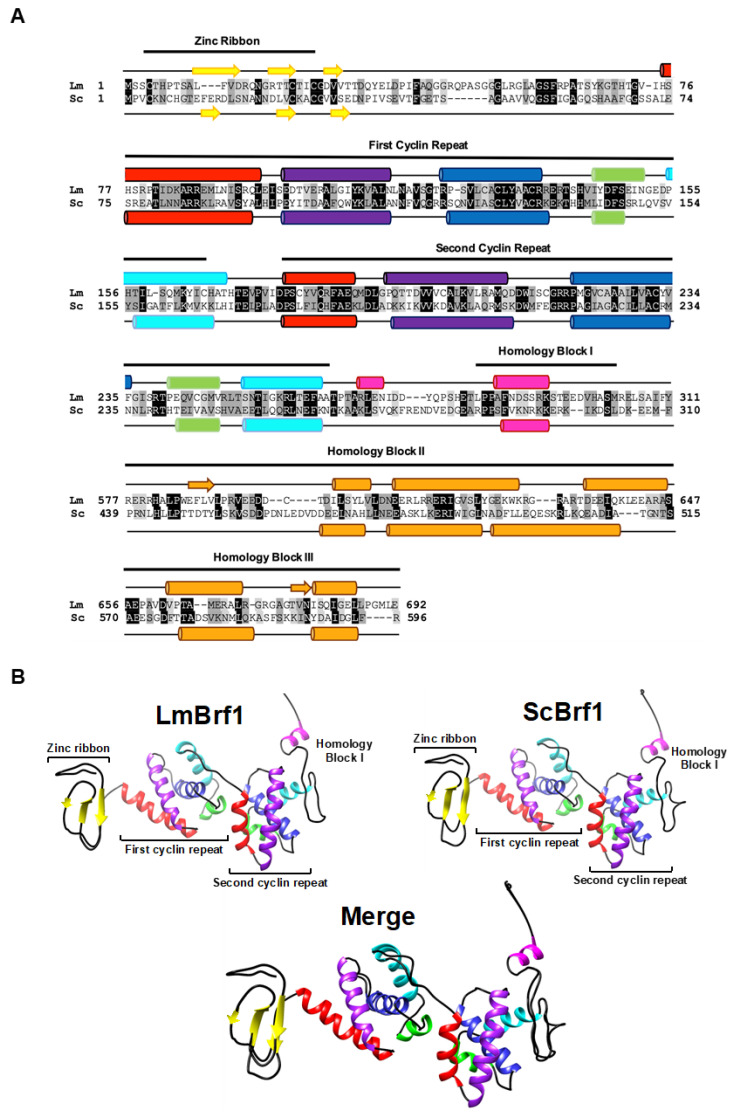
Sequence and predicted structure analyses of LmBrf1. (**A**) Sequence alignment of the N-terminal region and homology blocks I–III of TFIIB-related factor 1 (Brf1) from *Leishmania major* (Lm, LmjF.25.0440) and *Saccharomyces cerevisiae* (Sc, CAA68968.1). Please note that the last two lines of the alignment (Homology Block II and Homology Block III) are not contiguous with each other and with the rest of the alignment. Conservation is denoted by black shading, conserved substitutions are indicated by dark-gray shading, and semiconserved substitutions are denoted by light-gray shading, according to the ClustalΩ program. The zinc ribbon, both cyclin repeats, and the Brf1 homology blocks I, II, and III are indicated. Predicted secondary structure elements are shown for *L. major* (above the sequence) and for *S. cerevisiae* (below the sequence). The β-strands are denoted by yellow arrows, and α-helices by rounded rectangles. The five α-helices of the first and the second cyclin repeats are shown in the same colors. (**B**) Predicted three-dimensional structure of the N-terminal region of LmBrf1. Homology modeling was performed for LmBrf1 using the crystal structure from ScBrf1 as a template. A merge figure is also shown. All the structures are shown in the same colors presented in panel A. The quality of the models was estimated with Mod Eval server, showing a score of 0.70 (0.89 if we compare only the first and second cyclin repeats).

*LmjF.25.0440* was previously identified as the *L. major* ortholog of Brf1 (LmBrf1) [35]. A sequence comparison between LmBrf1 and its ortholog in *Saccharomyces cerevisiae* (ScBrf1) showed a relatively low sequence identity of 20%. Similarity was slightly higher in the N-terminal half of the protein (30%) than in the C-terminal half (18%). Nevertheless, the in silico analysis demonstrated that LmBrf1 contains the three conserved domains located in the N-terminal half of the protein: a zinc ribbon motif and two imperfect cyclin repeats (also known as TFIIB-related repeats) (Figure 1A). Moreover, LmBrf1 also contains moderately conserved Homology Blocks I, II, and III, which are characteristic of the C-terminal region of Brf1 from yeast species and human (Figure 1A) [45]. Notably, the predicted size of the LmBrf1 (77.2 kDa) is more than 10 kDa larger than ScBrf1 (66.9 kDa), due to insertions in the C-terminal region of the former. These insertions are present in other species of *Leishmania*, but absent in *T. cruzi* and *T. brucei* (Figure S1, Supplementary Materials).

The zinc ribbon fold in ScBrf1 consists of three antiparallel β strands [46]. Similarly, the zinc-binding domain in LmBrf1 is predicted to fold into three β strands, while the rest of the protein is mainly composed of α-helices (Figure 1A). Each cyclin repeat in LmBrf1 is predicted to fold into five α-helices (Figure 1A), as reported in other organisms [18,47]. The structure of LmBrf1 was further analyzed by generating the hypothetical three-dimensional structure of the N-terminal region (zinc ribbon, cyclin repeats, and homology block I) by homology modeling, using the crystal structure of ScBrf1 as a template. As shown in Figure 1B, the zinc ribbon in LmBrf1 exhibits the typical structure of the three antiparallel β strands, and the cyclin repeats present the characteristic globular organization of the five α-helices. Moreover, the hypothetical architecture of homology block I, folded into one helix, is conserved in LmBrf1 (Figure 1B). Thus, the predicted structure of LmBrf1 is similar to that of ScBrf1.

### 3.2. LmBRF1 Is a Nuclear Protein

**Figure 2:**
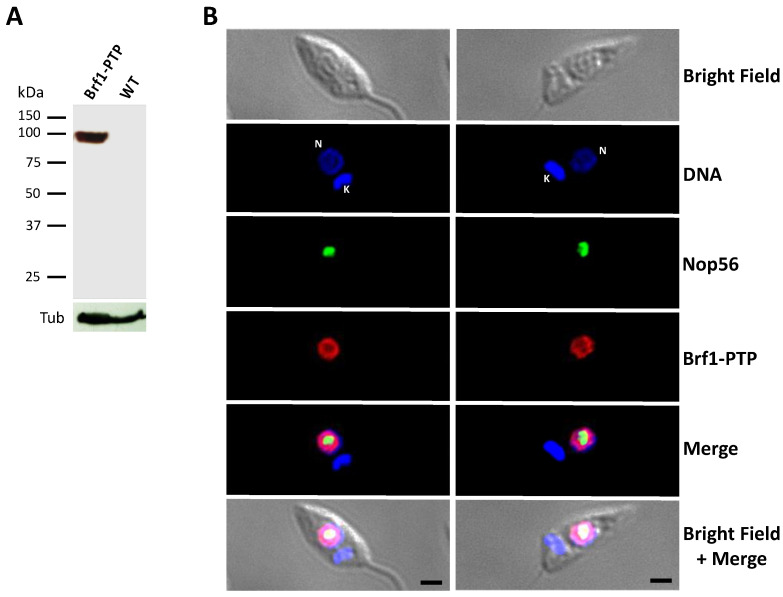
LmBrf1 is a nuclear protein. (**A**) Western blot analysis with cells that express the recombinant protein LmBrf1-PTP (Brf1-PTP (Prot C-TEV-Prot A)) and wild-type cells (WT). Membranes were incubated with an antibody against Prot C, which recognizes the PTP tag. As a loading control, a human α/β-tubulin antibody was used. (**B**) PTP-tagged LmBrf1 was detected by immunofluorescence with a rabbit anti-Prot C polyclonal antibody and an anti-rabbit immunoglobulin G (IgG) coupled with Alexa Fluor 568 (red). Nop56 was detected with a mouse anti-Nop56 antibody and an anti-mouse IgG secondary antibody conjugated with Alexa Fluor 488 (green). Nuclei (N) and kinetoplast (K) DNA were stained with 4’,6-diamidino-2-phenylindole (DAPI; blue). Scale bar indicates 2 μm.

To examine the subcellular localization of LmBrf1, a cell line where this protein is labeled with a C-terminal PTP tag was obtained to carry out indirect immunofluorescence assays. This tag is composed of Protein A (Prot A) and Protein C (Prot C) epitopes separated by a cleavage site of the TEV protease [43]. The expression of the LmBrf1-PTP protein was confirmed by Western blot analysis with an anti-Prot C antibody, showing a protein of ~97 kDa (expected from the fusion of the 20 kDa PTP tag to the ~77 kDa LmBrf1) (Figure 2A). The same antibody was used for the immunofluorescence experiments on fixed and permeabilized parasites. As shown in Figure 2B, LmBrf1 is localized in the nucleus of the parasites, as anticipated for a transcription factor. The nuclear distribution of LmBrf1 is very similar to that of LmBdp1 [33]. Notably, a small fraction of LmBrf1 seems to colocalize with the nucleolar protein Nop56 in the periphery of the nucleolus (in yellow in the Merge panels of Figure 2B).

### 3.3. The Half-Lives of the mRNAs from LmBrf1 and Other Components of the Pol III Transcription Machinery Are Very Similar

**Figure 3:**
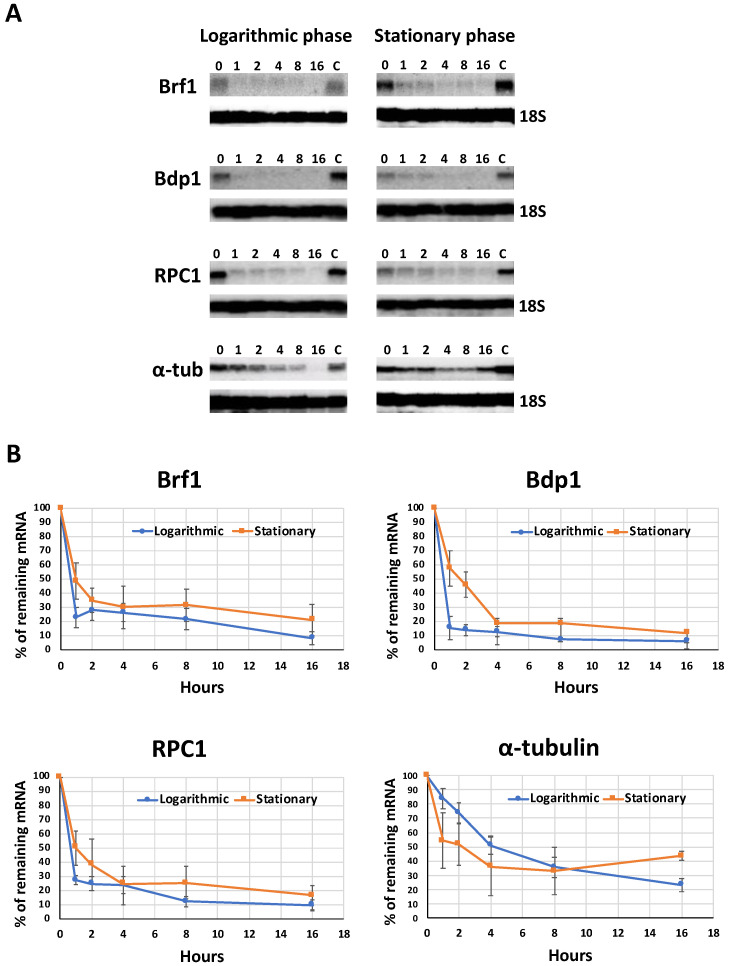
Half-lives of messenger RNAs (mRNAs) from Brf1, B double prime 1 (Bdp1), and RPC1. (**A**) Northern blots with total RNA from cells treated with actinomycin D (10 µg/mL) to inhibit transcription. RNA was isolated from logarithmic phase or stationary phase parasites at several time points (0, 1, 2, 4, 8, and 16 h) after addition of the drug. As a control, RNA from cells grown in the presence of dimethyl sulfoxide (10 µL/mL of culture), the solvent of actinomycin D, was included (lanes C). Hybridizations were performed with radioactive probes that corresponded to Brf1, Bdp1, RPC1, and α-tubulin. As loading control, membranes were re-hybridized with an 18S ribosomal RNA (rRNA) probe. (**B**) mRNA decay curves. Signals from the experiment shown in panel A and from two independent experiments were quantified and plotted. Results are presented from logarithmic-phase (blue line) and stationary-phase (red line) parasites. Error bars represent standard deviations.

In order to determine the decay rate of the LmBrf1 mRNA, Northern blot experiments were performed with total RNA from mid-log parasites that were grown in the presence of actinomycin D (10 mg/mL) for different times (between 0 and 16 h). The half-life of the LmBrf1 mRNA was around 39 min (Figure 3). Notably, the half-lives of the mRNAs from LmBdp1 (other TFIIIB subunit) and LmRPC1 (the largest Pol III subunit) were ~36 min and ~40 min, respectively (Figure 3). Thus, the decay rate of these three proteins involved in Pol III transcription is very similar. In contrast, a much longer half-life of ~4.3 h was observed for the α-tubulin mRNA (Figure 3). Similar experiments performed with stationary phase parasites showed that the half-lives of LmBrf1, LmBdp1, and LmRPC1 were ~60 min, ~72 min, and ~62 min, respectively (Figure 3, right panels). Thus, an increase in the half-lives of all three mRNAs was observed in stationary-phase promastigotes. This is different from the α-tubulin mRNA, whose half-life was reduced to ~2.25 h in nonproliferative cells (Figure 3).

### 3.4. Promastigotes Need at Least One Copy of the LmBrf1 Gene to Survive 

To study the role of LmBrf1 on cell growth and transcription in *L. major*, we planned to produce LmBrf1-null mutant parasites by replacing the endogenous genes with selectable marker genes. Since *L. major* Friedlin is a diploid organism [48], two consecutive rounds of targeted gene disruption were intended to generate double-knockout parasites. To fulfill this purpose, we produced plasmids pΔLmBrf1-pac and pΔLmBrf1-hyg, where the *puromycin* (*pac*) and *hygromycin* (*hyg*) resistance genes were bounded by 5′ and 3′ flanking regions of LmBrf1. 

**Figure 4:**
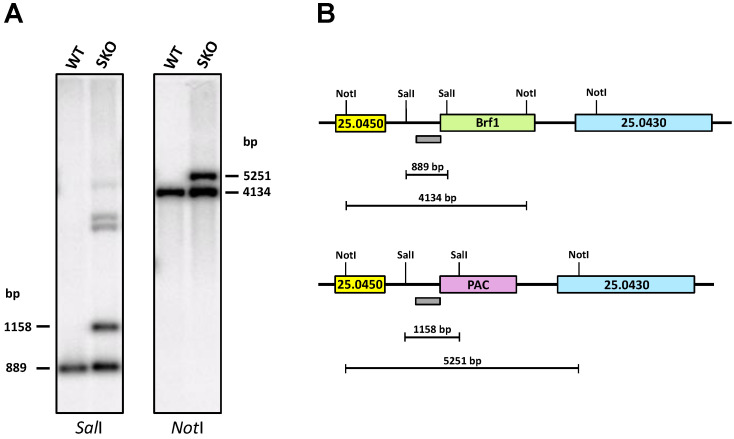
Southern blot analysis of LmBrf1 single-knockout parasites. (**A**) Genomic DNA from a single-knockout clone (SKO) and wild-type parasites (WT) was digested with *Sal*I and *Not*I. The probe corresponded to the 5′-targeting region of the LmBrf1 gene. The size of the observed bands is indicated. The larger fragments observed with DNA from the SKO clone digested with *Sal*I correspond to partial digestion of the enzyme. (**B**) Restriction maps of the wild-type LmBrf1 locus (top map) and mutant loci with a copy of LmBrf1 replaced with the *pac* gene (bottom map). Sizes of predicted restriction fragments after digestion with *Sal*I and *Not*I are shown. The location of the fragment employed as a probe is indicated with a gray bar.

The single-knockout cell line for LmBrf1 was generated by transfecting wild-type promastigotes with the targeting cassette from pΔLmBrf1-pac, and clones were selected with puromycin. The correct substitution of one LmBrf1 allele with the *pac* gene was demonstrated in one of the clones by Southern blot analysis using the 5′-targeting region as a probe, as the expected bands of ~1158 and ~5251 bp were observed when digesting with *Sal*I and *Not*I, respectively (Figure 4). The bands of the remaining endogenous LmBrf1 gene were also observed: ~889 bp with *Sal*I and 4134 bp with *Not*I (Figure 4). Thus, these results demonstrate that one copy of LmBrf1 was replaced with the *pac* gene. It is worth noting that the single-knockout cell line did not show any growth defect in relation to wild-type cells.

To generate the double-knockout cell line for LmBrf1, the single-knockout clone was transfected with the targeting cassette from vector pΔLmBrf1-hyg, and the culture was selected with both puromycin and hygromycin. However, we were unable to generate null mutants of LmBrf1, as all the transfected cultures died during the selection process. To verify that the hyg cassette functions properly, it was used to transfect wild-type promastigotes, and clones were successfully obtained in media with hygromycin. The correct substitution of one LmBrf1 allele with the *hyg* gene was shown by PCR analyses. Therefore, single-knockout parasites could be generated with both cassettes. This culture was electroporated with the pac cassette, but we did not obtain transfected cells, again. Thus, these results suggest that promastigotes of *L. major* require at least one copy of the LmBrf1 gene in order to survive. 

As an alternative procedure, the cell line that expresses the LmBrf1-PTP recombinant protein was transfected with the targeting cassette from pΔLmBrf1-hyg, and LmBrf1 single-knockout parasites were obtained after selecting with hygromycin. This cell line was then transfected with the pac cassette to try to generate the double-knockout parasites. However, we could not delete the second endogenous copy of LmBrf1, perhaps due to technical reasons that could have prevented the efficient transfection and/or drug selection of the electroporated parasites. Alternatively, it is possible that the expression of the recombinant LmBrf1-PTP protein was not high enough to allow the removal of the second allelic copy of LmBrf1, or that the presence of the PTP-tag might have reduced the activity of LmBrf1. Subsequent complementation experiments with new vectors will help to clarify this issue. 

### 3.5. LmBrf1 Binds to Pol III Promoters and Other Genomic Regions

The in vivo association of LmBrf1 to Pol III promoters was analyzed by ChIP experiments, using the *L. major* cell line that expresses LmBrf1 with a C-terminal PTP fusion. Chromatin was precipitated with a ChIP-grade anti-Prot A antibody that recognizes the two Prot A domains present in the PTP tag [49], and with a nonspecific mouse immune serum as negative control. The binding of LmBrf1-PTP to the purified DNA was evaluated by qPCR assays.

**Figure 5:**
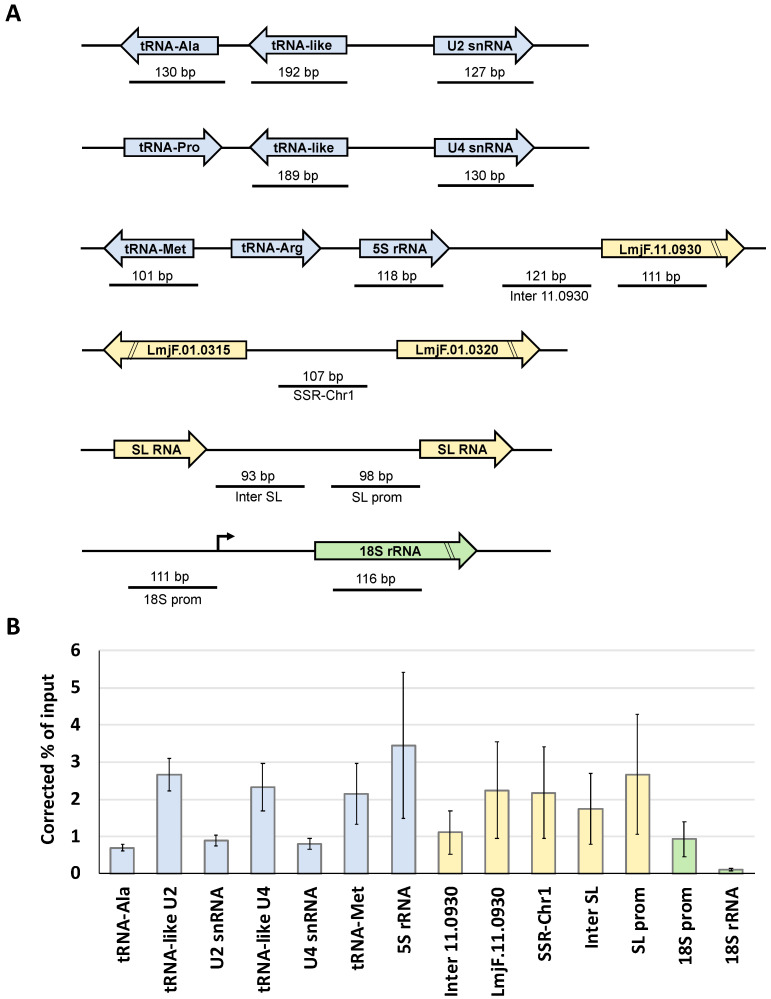
Chromatin immunoprecipitation (ChIP) analysis of LmBrf1. (**A**) Schematic representation of the genes and promoter regions examined. Genes transcribed by Pol III, Pol II, and Pol I are shown in blue, yellow, and green, respectively. Maps are not to scale. (**B**) Chromatin from a clonal cell line that expresses the recombinant protein LmBrf1-PTP was precipitated with a ChIP-grade anti-Prot A antibody. Precipitated DNA was analyzed by qPCR. The results from three independent ChIP experiments, each analyzed by two qPCR reactions, are shown. Results are presented as percentage of input, corrected by subtracting corresponding values from negative control precipitations performed with a nonspecific antiserum. Error bars indicate standard errors.

Results obtained from three independent experiments demonstrated that LmBrf1 associates with the tRNA-like and, to a lesser extent, to the tRNA-Ala, which are required for the expression of the U2 snRNA gene (Figure 5). Enrichment was also detected within the U2 snRNA gene, whose first nucleotides are needed for Pol III transcription of the gene [27]. Similar results were observed in the U4 snRNA locus, as high enrichment was detected in the upstream tRNA-like region, and lower association was found in the U4 snRNA gene (Figure 5). We also analyzed a genomic region from chromosome 11 that contains a 5S rRNA gene and two tRNA genes (Met and Arg) embedded within a Pol II transcription unit. High association of LmBrf1 was observed in the 5S rRNA and the tRNA-Met gene. Interestingly, occupancy of LmBrf1 was found in the *LmjF.11.0930* gene (which encodes a hypothetical protein and is transcribed by Pol II) and in the intergenic region between this gene and the 5S rRNA gene (Inter 11.0930) (Figure 5). Unexpectedly, association of LmBrf1 was found in the SSR from chromosome 1 (which controls transcription of all the protein-coding genes located on this chromosome) [50] and the spliced-leader (SL) RNA loci (transcribed by Pol II), as well as in the promoter region of the rRNA unit (transcribed by Pol I) (Figure 5).

### 3.6. Proteins That Participate in Pol I, Pol II, and Pol III Transcription Were Co-Purified with LmBrf1-PTP 

**Figure 6:**
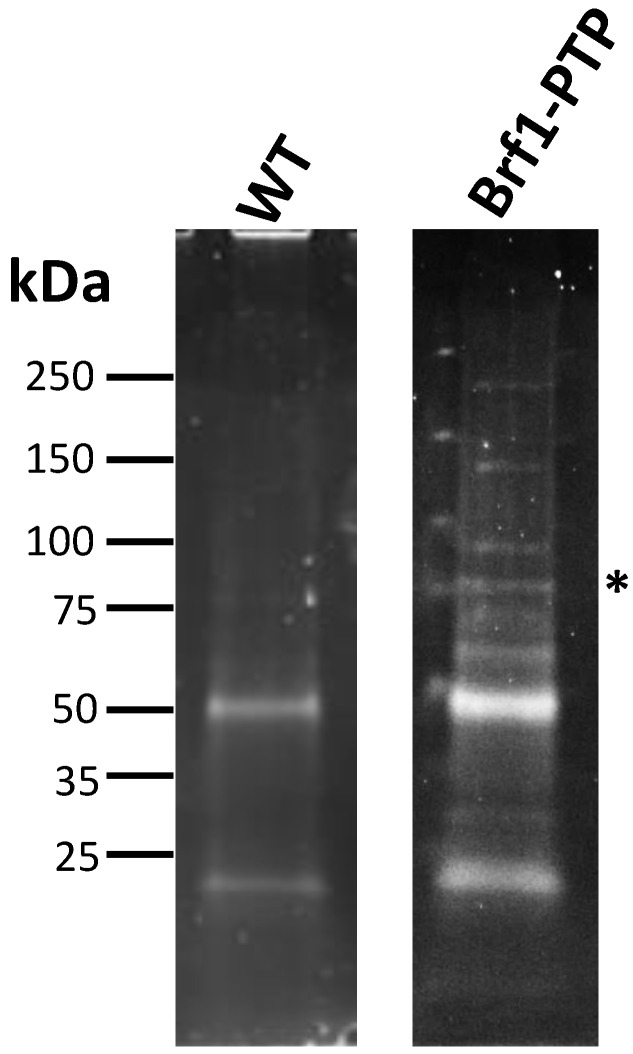
Tandem affinity purification with *L. major* cells expressing LmBrf1-PTP. SDS-PAGE of proteins co-purified with LmBrf1-PTP. The asterisk denotes the recombinant protein. A control experiment with wild-type cells (WT) is also shown. The samples were analyzed in 4–15% Mini-PROTEAN Precast Protein Gels stained with SYPRO Ruby.

To identify proteins that interact with LmBrf1, tandem affinity purification experiments were performed with the cell line that expresses the PTP-tagged version of LmBrf1. The correct expression of LmBrf1-PTP was confirmed by Western blotting and immunofluorescence (Figure 2). LmBrf1-PTP and associated proteins were purified, in successive steps, by IgG affinity chromatography, TEV protease elution, and anti-Prot C affinity chromatography. The purified proteins were eluted from the anti-Prot C column with a buffer that contains EGTA, and they were concentrated and analyzed by SDS-PAGE (Figure 6). Several different bands were observed, including one whose size corresponds to the tagged LmBrf1 (~81 kDa after TEV digestion, labeled with an asterisk in Figure 6). In the control experiment (mock purification using wild-type extract), some bands were also detected, which were identified by mass spectrometry as human keratins, bovine serum albumin, and multiple *L. major* ribosomal proteins, heat-shock proteins, translation elongation factors, actin, and α- and β-tubulins. These proteins are common contaminants in tandem affinity purifications [51].

In addition to LmBrf1, mass spectrometry analysis of the LmBrf1-PTP sample allowed the identification of multiple proteins that were divided in seven categories: transcription factors, Pol subunits, regulators of transcription and/or chromatin remodelers, DNA- or RNA-binding proteins, DNA replication, kinases or phosphatases, and other functions (Table 1). As anticipated, TBP was identified as one of the putative LmBrf1 partners. Nevertheless, Bdp1 (the third TFIIIB subunit) did not co-purify with LmBrf1-PTP. Notably, a putative ortholog of the TFIIIC subunit Tau131 was identified (*LmjF.12.0560*) (Table 1). This is a tetratricopeptide repeat protein annotated as hypothetical in the *L. major* genome database; however, sequence analysis with the HHpred server identified it as a probable ortholog of yeast Tau131 (E-value of 1.1 × 10^−41^). Pol III subunits RPC1, RPAC1 (shared with Pol I), and RPB5 (shared with Pol II) were also identified. Moreover, La protein, which binds to Pol III transcripts, co-purified with LmBrf1-PTP.

Interestingly, several proteins related to Pol II transcription were also purified. These include Pol II subunits RPB1 and RPB2, transcription elongation factors TFIIS and SPT6, some subunits from the PAF1 and the CCR4-NOT complexes, and FACT complex subunit SPT16 (Table 1). Moreover, several proteins involved in Pol I transcription were also identified, including Pol I subunits RPA1 and RPB5z, and high mobility group protein TDP1. 

## 4. Discussion

In this work, we characterized TFIIIB subunit Brf1 in the early-branching eukaryote *L. major*. Bioinformatic analyses demonstrated that the N-terminal half of LmBrf1 contains the distinctive zinc ribbon, cyclin repeats, and homology block I (Figure 1). Three-dimensional structure predictions show that the zinc ribbon folds into three antiparallel β strands, and each cyclin repeat folds into five α-helices with a globular organization. Homology block I is predicted to fold into a helix that is conserved in ScBrf1 (Figure 1). Notably, relatively conserved homology blocks II and III were identified in LmBrf1 (Figure 1). These two conserved sequences are characteristic of yeast species, and they are involved in the interactions of Brf1 with TBP and Bdp1 [23,24,45]. Homology blocks II and III were not identified previously in Brf1 from *T. brucei* [35]; however, sequence alignments indicated that they are actually present in this parasite, as well as in all the other trypanosomatids that were here analyzed (Figure S1, Supplementary Materials). These two homology blocks represent the most conserved sequences in the C-terminal half of Brf1 in trypanosomatids (Figure S1, Supplementary Materials). 

Immunofluorescence assays showed that LmBrf1 is a nuclear protein (Figure 2). A similar punctate nuclear signal was reported for other Pol III transcription factors in *L. major* and *T. brucei*, including Bdp1 [33] and Maf1 [41], as well as for the *L. major* 5S rRNA genes [29]. Even though the vast majority of LmBrf1 signal is nucleoplasmic, a small part of the immunostaining colocalized with Nop56 in the nucleolar periphery, which could support the association of LmBrf1 with the rRNA transcription unit that was found in ChIP experiments (Figure 5) and the possible interaction with proteins involved in Pol I transcription (Table 1), as discussed below. 

The fact that we were unable to obtain LmBrf1-null mutants suggests that this protein is essential for the viability of *L. major* promastigotes. In support of this possibility, inducible knockdown experiments in *T. brucei* procyclic forms demonstrated that Brf1 is indispensable for cell survival [35]. In other organisms, including yeast, Brf1 is an essential protein [52]. Similarly, LmBdp1 is an essential molecule for the viability of *L. major* promastigotes [33]. Other strategies, such as CRISPR/Cas9 genome editing, should be performed to demonstrate the essentiality of LmBrf1 for *L. major* growth.

Cellular RNA levels are determined through the interplay among RNA synthesis, processing, and degradation [53]. As Pol II transcription is constitutive in trypanosomatids, mRNA abundance is mainly regulated through processing by trans-splicing and polyadenylation, and by degradation [54]. It has been shown in mammals and *T. brucei* that the mRNAs encoding proteins with similar functions share comparable decay rates [53,54]. In line with these findings, our Northern blot analyses showed that the mRNAs from LmBrf1, Bdp1, and RPC1 (all involved in Pol III transcription) have a half-life of ~40 min, while the transcript from α-tubulin presents a half-life of ~4.3 h (Figure 3). These decay rates are relatively short in relation to human mRNAs, which show half-lives of 7 to 10 h [55]. However, even shorter decay rates have been reported in *T. brucei*, where the median mRNA half-life is about 20 min [54]. 

Compared to logarithmic-phase cells, in stationary-phase parasites, we observed an increase in the half-lives of the mRNAs encoding LmBrf1 (from 39 to 60 min), Bdp1 (from 36 to 72 min), and RPC1 (from 40 to 62 min) (Figure 3). The stabilization of mRNAs during the stationary phase was observed in *T. cruzi*, where it was suggested the presence of a regulation mechanism that protects mRNAs from degradation in stationary epimastigotes, as a strategy to perpetuate through this quiescent stage [56]. A similar mechanism might be present in nonproliferative promastigotes of *Leishmania*. Interestingly, unlike the other transcripts that we analyzed, the half-life of the α-tubulin mRNA was reduced in stationary phase cells from 4.30 to 2.25 h (Figure 3). Destabilization of α-tubulin transcripts in nonproliferative promastigotes has been reported in *Leishmania* [57,58]. Consequently, the stability of α-tubulin mRNA must be regulated by a different mechanism.

ChIP experiments demonstrated that LmBrf1 binds to tRNA, snRNA, and 5S rRNA genes (Figure 5). Similarly, TBP and Bdp1, the other subunits of TFIIIB, have been shown to bind to these genes in *L. major* [32,33]. ChIP results also indicated the unexpected binding of LmBrf1 to the promoter region of the rRNA genes (transcribed by Pol I), to the SSR from chromosome 1, the *LmjF.11.0930* gene, and to the SL RNA locus (transcribed by Pol II). The co-purification of proteins involved in Pol II and Pol I transcription with LmBrf1-PTP supports this finding (Table 1) (see below). Future genome-wide studies would reveal if LmBrf1 binds to other Pol II-dependent regions.

Tandem affinity purification experiments allowed the identification of several proteins that co-purified with LmBrf1-PTP, including TBP (Table 1). It was not surprising that we did not identify Bdp1, the third TFIIIB subunit, taking into consideration reports in other species that show that, while Brf1 interacts tightly with TBP and co-purifies, Bdp1 is weakly associated within the TFIIIB complex [59]. Nevertheless, it is also possible that the presence of the PTP-tag in LmBrf1 affected the interaction with Bdp1. Thus, other approaches should be employed to demonstrate the association between Bdp1 and Brf1 in *Leishmania*. Notably, a putative ortholog of TFIIIC subunit Tau131 (*LmjF.12.0560*) co-purified with LmBrf1-PTP. It is worth noting that our group identified the *T. brucei* ortholog (*Tb927.1.3860*) as an interacting partner of Pol III subunit RPC3 by tandem affinity purifications (manuscript in preparation). Thus, contrary to what was presumed [60], at least one subunit of TFIIIC is present in trypanosomatid parasites. Additionally, three Pol III subunits, the La protein, and a tRNA-binding domain-containing protein were co-purified with LmBrf1-PTP (Table 1). 

The multiple proteins identified by the mass spectrometry analyses (Table 1), including the proteins related to Pol II and Pol I transcription, represent potential interacting partners of LmBrf1-PTP. Future studies will help to determine if they actually associate, directly or indirectly, with LmBrf1. Among the Pol II-related proteins, we identified the transcription elongation factors TFIIS and SPT6, as well as two subunits of CCR4-NOT (Not1 and Not5), a complex that regulates gene expression at all steps, from transcription of mRNAs in the nucleus to their degradation in the cytoplasm [61,62]. We also identified four components of the PAF1 complex, which binds to Pol II to regulate transcriptional elongation and termination, as well as mRNA processing and export [63]. Interestingly, recent findings indicate that PAF1 also participates in Pol I and Pol III transcription [64]. Among the PAF1 subunits, a putative ortholog of RTF1 (HHpred E-value of 0.0011) was identified. Another protein that co-purified with LmBrf1-PTP is a RuvB-like DNA helicase, component of the INO80 chromatin remodeling complex that plays crucial roles in transcription by interacting with Pol II and TBP [65]. We also identified a putative Staphylococcal nuclease homolog/Tudor domain-containing protein (Table 1). These are evolutionarily conserved proteins involved in virtually all pathways of gene expression, from transcription to RNA silencing [66]. Regarding Pol I transcription, we identified Pol I subunits RPA1 and RPB5z, and the high-mobility group protein TDP1, which replaces histones on active Pol I transcription units in *T. brucei* to maintain an open chromatin structure and facilitate their transcription [67].

Several DNA- and RNA-binding proteins that could regulate gene expression were co-purified with LmBrf1-PTP, including a KD domain containing protein, putative ATP-dependent RNA helicase SUB2, and ALBA-domain protein 3 (Table 1). Protein phosphatase 2C and several protein kinases, whose role in transcription regulation has been extensively studied in other organisms [68,69,70], were also identified. Some factors that participate in DNA replication were also found (Table 1), which might suggest that DNA replication is coupled to transcription in *L. major*, as has been reported in human cells [71]. A reduced number of peptides was identified for some of the putative interacting partners (Table 1), which may indicate a weak and/or transient interaction with LmBrf1.

At the present time, we do not know if the association of LmBrf1 with some genes transcribed by Pol I and Pol II (Figure 5) and with proteins involved in Pol I and Pol II transcription (Table 1) has a functional significance. Several reports have demonstrated that, rather than functioning independently, the different types of RNA polymerases and their transcription factors can frequently work with one another to globally regulate and coordinate gene expression [72,73,74,75,76]. Therefore, it is possible that LmBrf1 helps to regulate transcription by all three RNA polymerases in *L. major*. Nevertheless, the interaction of LmBrf1 with Pol I- and Pol II-dependent regions and their transcription regulators might be fortuitous. A genome-wide study showed that TBP is highly enriched at most SSRs, the SL RNA genes, and the rRNA transcription units [32]. Thus, LmBrf1 could be attracted to these loci by TBP without playing a functional role. Future work will allow clarification of this subject. 

## 5. Conclusions

In this work, we showed that, despite sequence divergence, LmBrf1 possesses the six conserved domains of Brf1 orthologs. Moreover, the predicted three-dimensional structure of the N-terminal region of LmBrf1 is conserved. Our results also suggest that LmBrf1 is an essential nuclear protein. Northern blot assays showed that the mRNAs encoding LmBrf1, Bdp1, and RPC1 (involved in Pol III transcription) share similar half-lives. All three mRNAs were stabilized in stationary-phase parasites. ChIP experiments demonstrated that LmBrf1 associates to 5S rRNA, tRNA, and snRNA genes. However, unlike other species, LmBrf1 associates to the Pol I-dependent 18S rRNA gene promoter and to three regions transcribed by Pol II. Moreover, our results suggest the interaction of LmBrf1 with transcriptional complexes from all three RNA polymerases. Subunit Tau131 of TFIIIC was identified as one of the putative interacting partners of LmBrf1.

## Figures and Tables

**Table 1:** Proteins that co-purified with LmBrf1 ^a^.

TriTrypDB Name	Protein Function (Known or Putative)	Predicted Size(kDa)	Peptides ^b^
**Transcription factors**			
LmjF.19.1390	TATA-binding protein	31.1	43 (3)
LmjF.33.2810	Transcription elongation factor TFIIS	50.5	5 (1)
LmjF.12.0560	TFIIIC subunit Tau131, putative	149	1 (1)
LmjF.33.2820	TFIIS-like	63.9	1 (1)
LmjF.33.3090	Transcription elongation factor SPT6	158	1 (1)
**RNA polymerase subunits**			
LmjF.19.0660	RPAC1 (Pol I and Pol III)	47.3	6 (2)
LmjF.31.0160	RPB2 (Pol II)	133.8	6 (2)
LmjF.16.1350	RPA1 (Pol I)	199.7	4 (2)
LmjF.18.0780	RPB5 (Pol II and Pol III)	27.2	2 (2)
LmjF.34.0360	RPC1 (Pol III)	173.6	1 (1)
LmjF.18.0790	RPB5z (Pol I)	37.6	1 (1)
LmjF.31.2610	RPB1 (Pol II)	184.5	1 (1)
**Regulators of transcription and/or chromatin remodelers**			
LmjF.34.2610	RuvB-like DNA helicase, putative	53.6	11 (3)
LmjF29.0850	High-mobility group protein TDP1	33.6	8 (2)
LmjF.35.2850	PAF1 complex subunit LEO1	61.7	8 (2)
LmjF.29.1110	PAF1 complex novel subunit	68.4	7 (2)
LmjF.14.0890	PAF1 complex novel subunit (putative RTF1)	71.8	7 (3)
LmjF.19.0440	Nucleosome assembly protein, putative	39.7	5 (2)
LmjF.32.0950	Staphylococcal nuclease homolog/Tudor domain-containing protein, putative	102.3	5 (2)
LmjF29.0020	FACT complex subunit SPT16	114.7	4 (2)
LmjF.31.1750	Nucleosome assembly protein-like	45.4	3 (1)
LmjF.29.2340	Nucleosome assembly protein (NAP), putative	21.2	2 (2)
LmjF.29.2550	PAF1 complex subunit CTR9	96.6	2 (2)
LmjF.22.0450	Acetyltransferase (GNAT) family	32.3	1 (1)
LmjF.25.1840	CCR4-NOT transcription complex subunit Not5	72.4	1 (1)
LmjF.21.0800	CCR4-NOT transcription complex subunit Not1	248.7	1 (1)
**DNA or RNA binding proteins**			
LmjF27.1300	KH domain-containing protein, putative	59.9	13 (2)
LmjF.21.1552	ATP-dependent RNA helicase SUB2, putative	49.5	12 (3)
LmjF.21.0540	La protein homolog	37.2	11 (3)
LmjF.34.2580	ALBA-domain protein 3	22.6	6 (3)
LmjF.06.0010	Histone H4	11.4	6 (3)
LmjF.28.1530	ATP-dependent RNA helicase FAL1, putative	44	6 (3)
LmjF.07.1000	RNA-binding protein-like protein	35	5 (3)
LmjF.18.0700	HEAT repeats, putative	77.3	5 (3)
LmjF.30.3090	RNA-binding protein 42 (RNA-binding motif protein 42), putative	36.3	5 (2)
LmjF.30.3430	Protein Mkt1, putative	90.2	4 (2)
LmjF.12.1220	WD repeat and HMG-box DNA-binding protein, putative	150.2	4 (1)
LmjF.24.1490	WD domain, G-beta repeat, putative	47.5	3 (1)
LmjF.22.0470	tRNA-binding domain-containing protein	44.8	2 (1)
**DNA replication**			
LmjF.28.0850	DNA replication licensing factor MCM2, putative	110.4	7 (1)
LmjF.09.0250	DNA replication licensing factor MCM4, putative	97.2	5 (1)
LmjF27.0550	Replication factor C, subunit 4, putative	39.5	5 (2)
LmjF.15.1450	Proliferative cell nuclear antigen (PCNA), putative	32.4	4 (2)
LmjF.36.6710	Replication factor C subunit 3, putative	39.3	3 (2)
**Kinases or phosphatases**			
LmjF.32.2950	Nucleoside diphosphate kinase B	16.6	14 (3)
LmjF.25.0750	Protein phosphatase 2C	44.9	6 (3)
LmjF.10.0200	Mitogen-activated protein kinase 10, putative	46.3	2 (2)
LmjF.36.0550	Cdc2-related kinase 3	35.6	2 (1)
LmjF.34.2820	Regulatory subunit of protein kinase A-like	71.6	2 (1)
**Other functions**			
LmjF.08.1110	Stress-induced protein STI1	62.1	20 (3)
LmjF.36.3210	14-3-3 protein 1, putative	29.7	15 (3)
LmjF.17.0870	META domain-containing protein	48.4	8 (3)
LmjF.32.2150	Hypothetical protein, conserved	117.6	8 (3)
LmjF.21.0430	Hypothetical protein, conserved	44.7	8 (2)
LmjF.36.2510	Nucleoporin NUP96	96.9	8 (1)
LmjF.35.0070	Prohibitin, putative	32.3	6 (2)
LmjF.21.1555	Hypothetical protein, conserved	46.4	5 (3)
LmjF.13.1360	Hypothetical protein, conserved	90.9	5 (3)
LmjF.32.0840	Hypothetical protein, conserved	57.4	4 (3)
LmjF.33.2270	Hypothetical protein, conserved	59.6	3 (2)
LmjF.32.0620	Hypothetical protein, conserved	41.8	3 (1)
LmjF.09.0840	Uncharacterized protein family (UPF0160), putative	42.5	3 (1)
LmjF.33.2800	Hypothetical protein, conserved	17	2 (2)
LmjF.22.0500	Hypothetical protein, conserved	54.9	2 (2)

^a^ Proteins likely to be contaminants (including multiple ribosomal proteins, translation factors, tubulins, heat-shock proteins, mitochondrial proteins, and aminoacyl-tRNA synthetases) were not included. ^b^ The number of different peptides detected in three tandem affinity purification experiments is indicated. The number in parenthesis shows the number of experiments in which peptides were detected.

## Data Availability

All data generated or analyzed in this study are available from the corresponding author on reasonable request.

## Supplementary Materials

The following are available online at https://www.mdpi.com/2073-4425/12/2/280/s1: Figure S1. Brf1 orthologs in trypanosomatids. Multiple sequence alignment of the Brf1 proteins from *L. major* (Lmj), *L. mexicana* (Lmx), *L. braziliensis* (Lbr), *L. tarentolae* (Ltr), *L. donovani* (Ldv), *T. brucei* (Tbr), and *T. cruzi* (Tcr). Conservation is denoted by black shading, conserved substitutions are indicated by dark-gray shading, and semiconserved substitutions are denoted by light-gray shading, according to the ClustalΩ program. The zinc ribbon, first and second cyclin repeats, and the Brf1 homology blocks I, II, and III are indicated.
