# Supplementary material for: Participation of TFIIIB Subunit Brf1 in Transcription Regulation in the Human Pathogen Leishmania major
Source: Genes (Basel). 2021 Feb 16;12(2):280. doi: 10.3390/genes12020280 (PMC7920299; doi:10.3390/genes12020280)
Supplement: Supplementary file 1 [file genes-12-00280-s001.pdf]

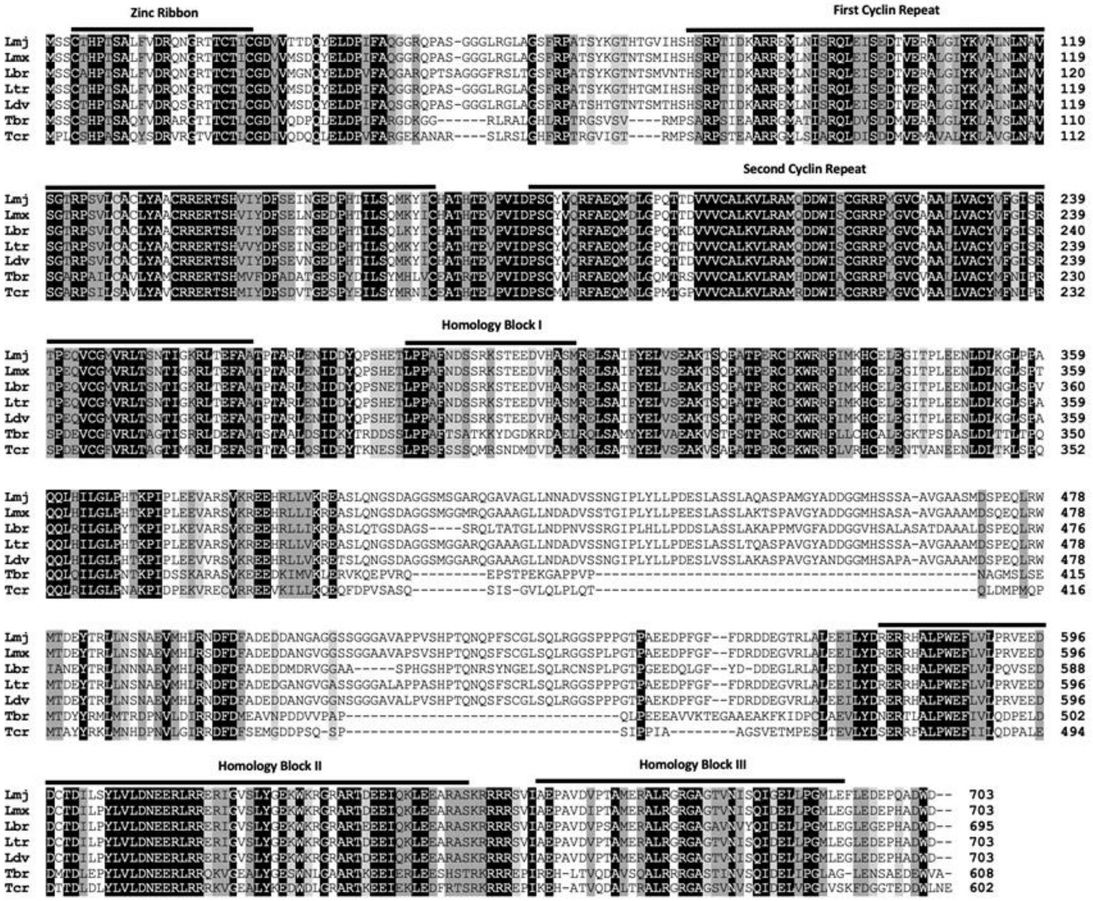
Zinc Ribbon
First Cyclin Repeat
Second Cyclin Repeat
Homology Block I
Homology Block II
Homology Block III
Lmj
Lmx
Lbr
Ltr
Ldv
Tbr
Tcr
